# Involvement of nNOS, and α1, α2, β1, and β2 Subunits of Soluble Guanylyl Cyclase Genes Expression in Anticonvulsant Effect of Sumatriptan on Pentylenetetrazole-Induced Seizure in Mice

**DOI:** 10.22037/ijpr.2020.112594.13844

**Published:** 2020

**Authors:** Faiza Mumtaz, Hamed Shafaroodi, Sadaf Nezamoleslami, Muhammad Zubair, Mohammad Sheibani, Vahid Nikoui, Mahmoud Ghazi-Khansari, Ahmad Reza Dehpour

**Affiliations:** a *Experimental Medicine Research Center, Tehran University of Medical Sciences, Tehran, Iran.*; b *Department of Pharmacology, School of Medicine, Tehran University of Medical Sciences, Tehran, Iran. *; c *Key Laboratory of Integrated Management of Crop Diseases and Pests, College of Plant Protection, Nanjing Agriculture University, Nanjing, 210095, PR China. *; d *Razi Drug Research Center, Iran University of Medical Sciences, Tehran, Iran.*

**Keywords:** Sumatriptan, Pentylenetetrazole, Nitric oxide, Soluble guanylyl cyclase, Cyclic guanosine monophosphate, Seizure, Mice

## Abstract

Epileptic seizure is phenomenon of abnormal synchronous neuronal discharge of a set of neurons in brain as a result of neuronal excitation. Evidence shows the nitric oxide (NO) involvement in neuronal excitability. Moreover, the role of cyclic guanosine monophosphate (cGMP) activation in seizure pathogenesis is well-established. Sumatriptan is a selective agonist of 5-Hydroxytryptamine1B/D auto-receptor, has been reassessed for its neuroprotection. This study was aimed to explore the anticonvulsant effect of sumatriptan through possible involvement of NO-cGMP pathway in mice. For this purpose, the protective effect of sumatriptan on PTZ-induced clonic seizure threshold (CST) was measured using NO-cGMP pathway inhibitors including N(G)-nitro-L-arginine (L-NNA, 1, 5, and 10 mg/kg), 7-nitroindazole (7-NI, 30, 45, and 60 mg/kg), aminoguanidine (AG, 30, 50, and 100 mg/kg), methylene blue (MB, 0.1, 0.5, and 1 mg/kg) and sildenafil (5, 10, and 20 mg/kg). The involvement of nitrergic system was further confirmed by measurement of nitrite levels by Griess reaction. The gene expression of neuronal nitric oxide synthase (nNOS) and subunits of soluble guanylyl cyclase (sGC) was studied using qRT-PCR analysis. Acute administration of sumatriptan (1.2 and 0.3 mg/kg) in combination with subeffective doses of NOS, sGC, and phosphodiesterase 5 inhibitors significantly reversed the PTZ-induced CST (*P ≤ *0.001). The nitrite level in prefrontal cortex was significantly attenuated by sumatriptan (*P* ≤ 0.01). Furthermore, sumatriptan downregulated the PTZ-induced mRNA expression of nNOS (*P* ≤ 0.01), α1 (*P* ≤ 0.001), α2 (*P* ≤ 0.05), and β1 (*P *≤ 0.05) genes in cerebral cortex of mice. In conclusion, the anticonvulsant activity of sumatriptan at least, in part, is mediated through inhibiting NO-cGMP pathway.

## Introduction

Epilepsy is an overwhelming neurological disorder worldwide, recognized by repetitive seizures through hyper neuronal discharge. Hence, epileptic seizure is phenomenon of abnormal neuronal excitation of a set of neurons in brain as a result of Ca^2 +^ influx (1). Already available anticonvulsants act through hyperpolarization, altering calcium and sodium channels, and modulating the activity of N-methyl-D-aspartate (NMDA) or AMPA/kainite receptors (2, 3). Postsynaptic Ca^2 +^ transfer through NMDA receptors cause subsequent activation of nitric oxide (NO) pathway (4). Whereas, among all three isoforms of nitric oxide synthase (NOS), the neuronal NOS (nNOS) is mainly responsible to generate NO in brain (5). Furthermore, the pentylenetetrazole (PTZ)-induced clonic seizure model is considered as an authentic experimental model, causing NO mediated neuro-excitation (6).

It is well-known that NO exerts proconvulsive properties in PTZ-induced seizure model. In contrast, the nitrergic system inhibitors are assumed to possess anticonvulsive properties against PTZ-induced seizure (7, 8). Moreover, in central nervous system (CNS), NO releases the cyclic guanosine monophosphate (cGMP) through stimulation of soluble guanylyl cyclase (sGC), an intracellular physiological receptor of NO (9). In different brain regions, sGC comprises of two subunits of α and β, which further exists in four isoforms namely α1, α2, β1, and β2, in form of two heterodimers α1/β1 and α2/β1 (10, 11). The role of NO-cGMP pathway in seizure pathogenesis is well-established (12). Hence, inhibitors of NO-cGMP pathway could elevate the PTZ-induced seizure threshold (13).

Sumatriptan is a selective agonist of 5-HT1B/1D auto-receptors, used as an excellent remedy to treat migraine and cluster headache. Although, the antimigraine effect of sumatriptan is mediated through 5HT1B/1D receptors, in CNS the involvement of NO and cGMP signaling pathway in pharmacological and clinical applications of sumatriptan has been well-documented in literature (14-18). Furthermore, the inhibitory role of sumatriptan on seizure threshold has been already reported through possible involvement of non-serotonergic 5-HT1B/D receptors and nitric oxide (NO) pathway (19-21).

There is little evidence about the involvement of cGMP signaling in the anticonvulsant effect of sumatriptan in experimental model of PTZ-induced seizure. Therefore, we aimed to evaluate the possible role of NO-cGMP signaling in anticonvulsant activity of sumatriptan. Furthermore, we investigated the involvement of nNOS, and α1, α2, β1, and β2 subunits of soluble guanylyl cyclase genes expression in anticonvulsant properties of sumatriptan in PTZ-induced clonic seizure in mice.

## Experimental

*Animals*

Adult male Naval Medical Research Institute (NMRI) mice weighing 23-30 grams were used in the study. The animals were housed in a temperature (22 ± 5 °C) and humidity (60-70%) controlled room maintained at 12 h light /dark cycles and had free access to ad libitum diet and water. The experimental procedures were in accordance with the National Institute of Health guide for the Care and Use of Laboratory Animals (8^th^ Edition, 2011, the National Academies Press, Constitution Ave., Washington, DC, USA) and approved by the research ethical committee of Tehran University of Medical Sciences.

*Chemicals*

The drugs used in the present study were: sumatriptan (5-HT1B/1D agonist), pentylenetetrazole (PTZ, GABA_A_ receptor antaggonist), aminoguanidine (AG, a specific iNOS inhibitor), 7-Nitroindazole (7-NI, a specific nNOS inhibitor), sildenafil (PDE5 inhibitor), sodium nitrite, and Trizol reagent, which were purchased from Sigma (St Louis, MO, USA). The other used chemicals were as below: N(G)-nitro-L-arginine (L-NNA, a non-specific NOS inhibitor, Fluka, Switzerland); methylene blue (MB, a sGC inhibitor, Hoechst, Germany); Griess reagent (Enzo life sciences, NY, USA); and cDNA synthesis kit (Life Technologies Ltd., UK).

*Drugs preparation and administration*

**Figure 1:**
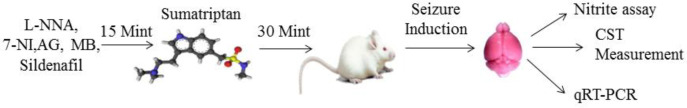
Schematic description of study design

All drugs were dissolved in normal saline except 7-NI, which was dissolved in 1% solution of Tween 80. All drugs were injected in a volume of 10 mL/kg via intraperitoneal (i.p.) rout except PTZ (0.5%), which was administered via intravenous (i.v.) route for all experiments. The dose selection, route of drug administration, and injection time of all drugs were selected by pilot study and previously published research work (22). In all experiments, based on pilot study sumatriptan was injected at dose rate of 0.3 and 1.2 mg/kg (0.3, 0.6, 1.2, 2.4 mg/kg) and 30 min prior to PTZ-induced seizures as reported previously (21). The drug administration schedule is represented in Figure 1.

*Study groups*

The following study groups were examined to determine the involvement of NO/cGMP pathway in anticonlvusant effect of sumatriptan. In group 1, mice were injected with the corresponding volume of saline (control) or vehicles (Tween 80) before PTZ-induced clonic seizure (CS). In group 2, mice were injected with different doses of L-NNA (1, 5, and 10 mg/kg), 7-NI (30, 45, and 60 mg/kg), AG (30, 50, and 100 mg/kg), MB (0.1, 0.5, and 1 mg/kg) and sildenafil (5, 10, and 20 mg/kg) alone. In group 3, mice were injected with L-NNA (1 mg/kg), 7-NI (30 mg/kg), AG (30, 50, and 100 mg/kg), MB (0.5 mg/kg) and sildenafil (5 mg/kg) along with sumatriptan. In group 4, mice were coadministered with 7-NI (30 mg/kg) and MB (0.5 mg/kg) along with sumatriptan.

*Seizure induction*

PTZ-induced seizures threshold was measured by previously studied protocol (23). In short, PTZ (0.5%, 5 mg/mL) was injected using a 30 gauge winged infusion set into the tail vein of freely moving mice at a constant rate of 1 mL/min. Infusion was stopped immediately when forelimb clonus followed by full clonus of the body, was used as the endpoint for PTZ-induced CST. The minimal dose of PTZ (mg/kg) required to induce a clonic seizure was measured as the index of CST. Following formula was used to measure PTZ-induced CST: [infusion duration (min) × infusion rate (mL/min) × PTZ concentration (mg/mL) × 1000]/[weight of mouse (g)]. All of the animals were sacrificed via cervical dislocation throughout the study.

*Nitrite assay*

Nitrite level was assessed as an index of NO production based on Griess reaction (24, 25). Prefrontal cortex (PFC) were dissected on ice-cold surface and immediately immersed in liquid nitrogen. Briefly, samples were homogenized in chilled phosphate buffer (pH 7.4) and centrifuged at 800 g for 20 min at 4 ℃ to obtain supernatant. Then, supernatant was mixed with equal volume of Griess reagent and incubated at room temperature for 30 min under reduced light. Concentration of nitrite was quantified using spectrophotometer at 540 nm against a nitrite standard (0.1 M NaNO_2_ in water).

*Quantitative real-time polymerase chain reaction (QRT-PCR) study*

The cerebral cortex tissues were dissected and freezed at-80 ºC till further analysis. The frozen tissues were homogenized and total RNA was extracted by Trizol reagent. Then, cDNA kit was used to obtain the single strand cDNA followed by amplification of specific mRNA. The specific primers used for PCR amplification are shown in Table 1. A LightCycler^®^96 system (Roche Diagnostics, Mannheim, Germany) was used to perform QRT-PCR. Thermal reaction conditions were maintained in accordance to previous studies (26). The relative expression of target genes was normalized to GAPDH expression in the same reaction. For calculations, 2-ΔΔCT method was used to measure the fold change in the mRNA expression of target genes as compared to control. All experiments run in triplicate and following formula was used to calculate: ΔΔCT = (CT target–CT GAPDH) experimental sample–(CT target–CT GAPDH) control sample.

*Statistical analysis*

GraphPad Prism 7 software (GraphPad Software San Diego, CA, USA) was used for statistical analysis. All data are expressed as mean ± standard error of the mean (SEM). The statistical differences between groups were analyzed by one-way analysis of variance (ANOVA) followed by Tukey’s *post-hoc* test. *P* ≤ 0.05 was considered as statistically significant.

## Results

*Dose response of sumatriptan on Clonic Seizure Threshold (CST)*

**Figure 2:**
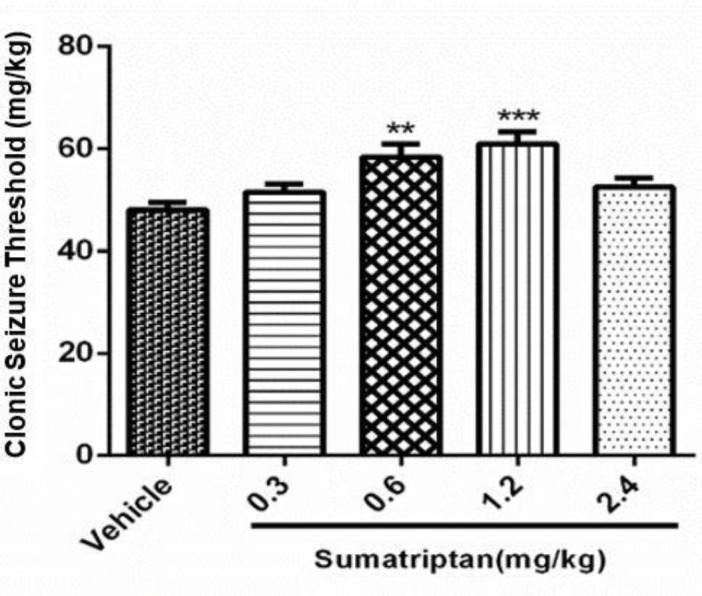
Effect of various doses of sumatriptan (0.3, 0.6, 1.2 and 2.4 mg/kg) on clonic seizure threshold (CST) on PTZ induced seizures in mice. Data are expressed as mean ± S.E.M. for 8 mices, analyzed by one-way ANOVA followed by Tukey's *post-hoc*-test. **P ≤ 0.01, ***P ≤ 0.001 compared to control/vehicle

The data in Figure 2 shows the effect of various doses of sumatriptan (0.3, 0.6, 1.2, 2.4mg/kg) on PTZ induced seizure. Statistical analysis showed that sumatriptan at doses of 0.6 mg/kg (*P* ≤ 0.01) and 1.2 mg/kg (*P* ≤ 0.001) significantly attenuated PTZ induced clonic seizure. All other doses remained non-significant (*P* > 0.05) as compared to control.

*Involvement of NO in anticonvulsant effect of sumatriptan*

**Figure 3:**
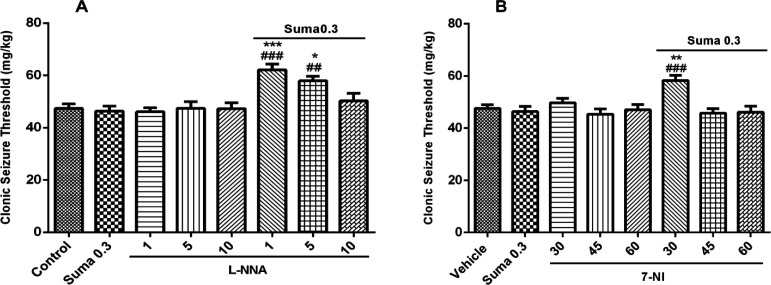
Effect of subeffective doses of NOS inhibitors (A) L-NNA (1, 5, and 10 mg/kg), (B) 7-NI (30, 45, and 60 mg/kg) alone or in combination with acute subef fective dose of sumatriptan (0.3 mg/kg) on PTZ-induced clonic seizure threshold (CST) in mice. Data are expressed as mean ± S.E.M. for 8 mices, analyzed by one-way ANOVA followed by Tukey's *post-hoc *test. *P ≤ 0.05, **P ≤ 0.01, ***P ≤ 0.001 compared to control/vehicle, ^##^*P* ≤ 0.01, ^###^*P* ≤ 0.001 compared to sumatriptan group

**Figure 4:**
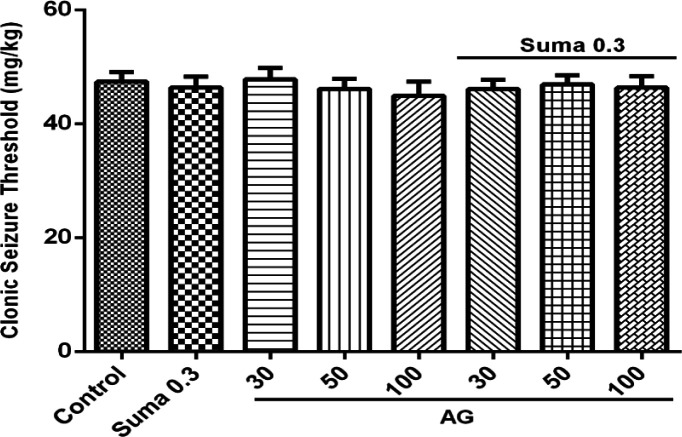
Effect of subeffective doses of AG (30, 50, and 100 mg/kg) alone or in combination with acute subeffective dose of sumatriptan (0.3 mg/kg) on PTZ-induced CST in mice. Data are expressed as mean ± S.E.M. for 8 mices, analyzed by one-way ANOVA followed by Tukey's *post-hoc *test

Involvement of NO in anticonvulsant effect of sumatriptan was confirmed using NOS inhibitors including L-NNA (a nonspecific NOS inhibitor) and 7-NI (neuronal NOS inhibitor). The results revealed that L-NNA and 7-NI alone had no effect on CST (*P* > 0.05). Figure 3A shows that pretreatment of mice with L-NNA (1 and 5 mg/kg, i.p.) 15 min before subeffective dose of sumatriptan (0.3mg/kg, i.p.) significantly increased the CST compared to sumatriptan treated group (*P* ≤ 0.001, *P* ≤ 0.01, respectively). Illustrating in Figure 3B, 7-NI (30 mg/kg, i.p.) 15 min prior subeffective dose of sumatriptan significantly boosted the CST compared to sumatriptan treated group (*P* ≤ 0.001). However, as Figure 4 shows, the pretreatment of mice with the selective iNOS inhibitor, AG (30, 50, and 100 mg/kg, i.p.) failed to augment the anticonvulsant effect of a subeffective dose of sumatriptan (*P* > 0.05). 

*Involvement of cGMP in anticonvulsant effect of sumatriptan*

**Figure 5:**
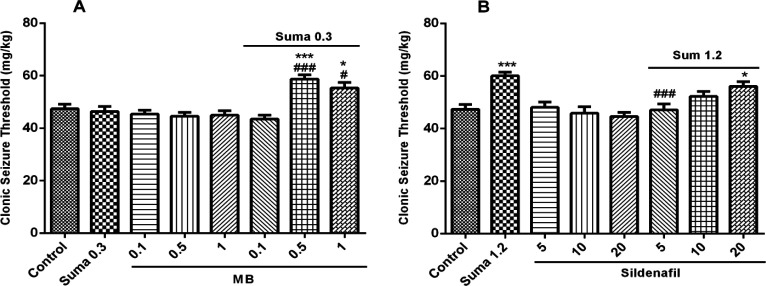
Effect of subeffective doses of (A) MB (0.1, 0.5, and 1 mg/kg), (B) sildenafil (5, 10, and 20 mg/kg) alone or in combination with acute subeffective and effective doses of sumatriptan (0.3 and 1.2 mg/kg) on PTZ-induced CST in mice. Data are expressed as mean ± S.E.M. for 8 mices, analyzed by one-way ANOVA followed by Tukey's *post-hoc *test. *P ≤ 0.005, ***P ≤ 0.001 compared to control, ^#^*P *≤ 0.05, ^###^*P* ≤ 0.001 compared to sumatriptan group

Involvement of cGMP in anticonvulsant effect of sumatriptan was confirmed using cGMP pathway inhibitors including MB (sGC inhibitor) and sildenafil (PDE5 inhibitor). The results showed that MB and sildenafil alone had no effect on CST *(P* > 0.05). Figure 5A illustrates that pretreatment of the mice with MB (0.5 and 1 mg/kg, i.p.) 15 min prior the subeffective dose of sumatriptan (0.3 mg/kg, i.p.) significantly increased the CST compared to sumatriptan treated group (*P* ≤ 0.001, *P* ≤ 0.05, respectively). Figure 5B shows that sildenafil (5 mg/kg, i.p.) 15 min before the effective dose of sumatriptan significantly diminished the CST compared to the sumatriptan treated group (*P* ≤ 0.001). 

*Interaction of NO and cGMP pathway*

**Figure 6:**
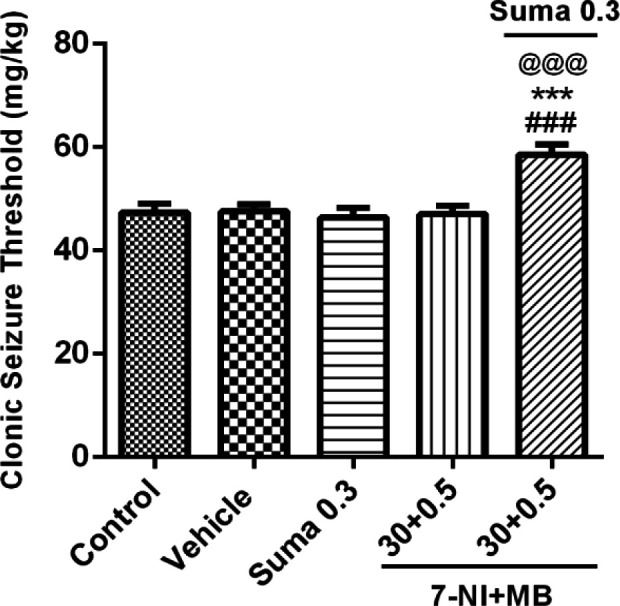
Effect of subeffective doses of coadministration of 7-NI (30 mg/kg) + MB (0.5 mg/kg) alone or in combination with acute subeffective dose of sumatriptan (0.3 mg/kg) on PTZ-induced CST in mice. Data are expressed as mean ± S.E.M. for 8 mices, analyzed by one-way ANOVA followed by Tukey's *post-hoc *test. ***P ≤ 0.001 compared to control group, ^@@@^*P* ≤ 0.001 compared to vehicle group, ^###^*P* ≤ 0.001 compared to sumatriptan group

To investigate the NO mediated activation of cGMP and their possible interaction, the subeffective doses of 7-NI (30 mg/kg, i.p.) + MB (0.5 mg/kg, i.p.) were coadministered alone or 15 min before subeffective dose of sumatriptan (0.3 mg/kg, i.p). Figure 6 shows that coadministration of 7-NI + MB with sumatriptan significantly boosted the CST compared to control/vehicle and sumatriptan treated groups (*P* ≤ 0.001).

*Effect of sumatriptan on NO metabolites*

**Figure 7:**
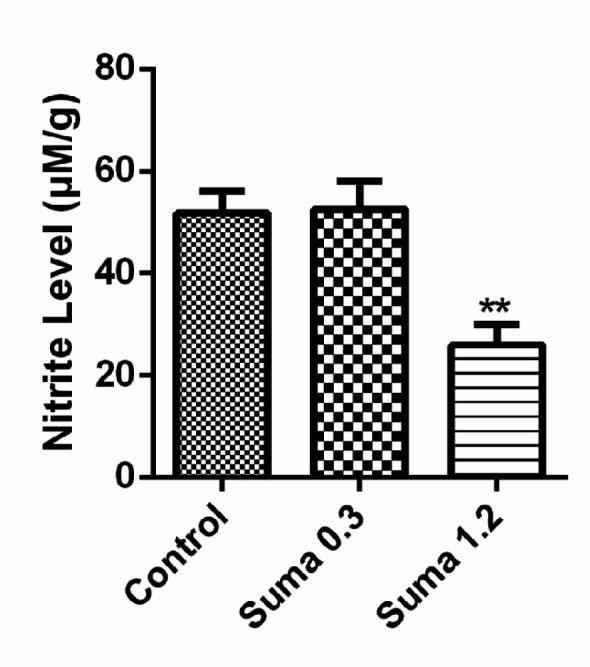
Effect of sumatriptan on prefrontal cortex (PFC) nitrite levels of mice. Data are expressed as mean ± S.E.M. for 4 mices, analyzed by one-way ANOVA followed by Tukey's *post-hoc *test. **P ≤ 0.01 compared to control group

As demonstrated in Figure 7, the effective dose of sumatriptan (1.2 mg/kg, i.p) significantly reduced the nitrite levels in prefrontal cortex (PCF) compared to the control group (*P* ≤ 0.01). 

**Figure 8:**
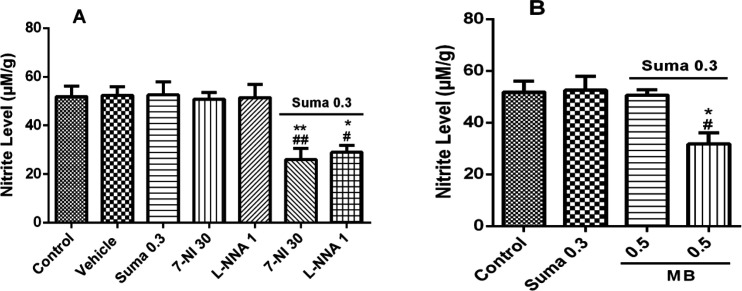
Effect of subeffective doses of (A) 7-NI (30 mg/kg) and L-NNA (1 mg/kg), (B) MB (0.5 mg/kg) with acute subeffective dose of sumatriptan (0.3 mg/kg) on PFC nitrite levels of mice. Data are expressed as mean ± S.E.M. for 3 mices, analyzed by one-way ANOVA followed by Tukey's *post-hoc *test. *P ≤ 0.05, **P ≤ 0.01, ***P ≤ 0.001 compared to control/vehicle group. ^#^*P *≤ 0.05, ^##^*P* ≤ 0.01, ^###^*P* ≤ 0.001 compared to sumatriptan group

Figure 8A shows that nitrite concentration was significantly decreased by subeffective dose of sumatriptan (0.3 mg/kg, i.p.) when treated with subeffective doses of L-NNA (1 mg/kg, i.p.) and 7-NI (30 mg/kg, i.p.) compared to control/vehicle (*P* ≤ 0.01, *P* ≤ 0.005, respectively). Figure 8B shows the nitrite concentration was significantly decreased by subeffective dose of sumatriptan (0.3 mg/kg, i.p.) when treated with subeffective dose of MB (0.5 mg/kg, i.p.) compared to the control group (*P* ≤ 0.005). 

*Evaluation of qRT-PCR genes expression*

**Figure 9:**
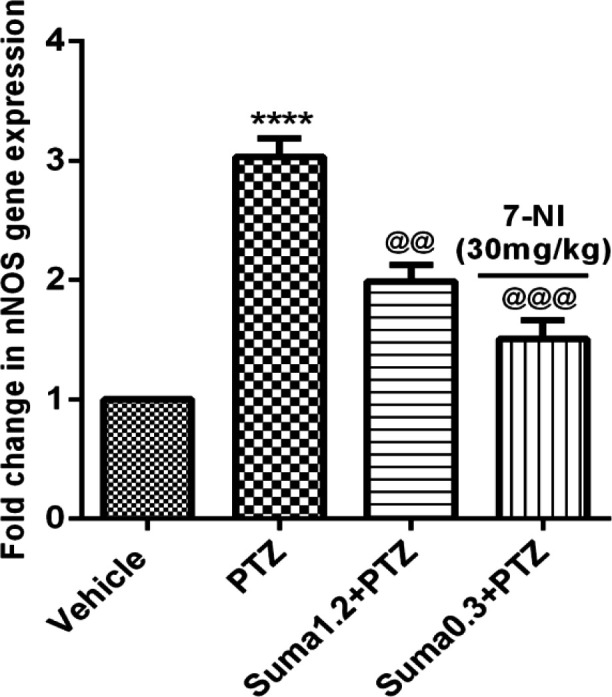
Effect of sumatriptan on relative mRNA expression of nNOS in cerebral cortex of mice against PTZ-induced clonic seizure. Data are expressed as mean ± S.E.M. for 3 mices, analyzed by one-way ANOVA followed by Tukey's *post-hoc *test. ****P ≤ 0.0001 compared to control group, ^@@^*P* ≤ 0.01, ^@@@^*P *≤ 0.001 compared to PTZ group

Figure 9 illustrates that PTZ administration significantly increased the mRNA expression of nNOS in cerebral cortex of mice (*P* ≤ 0.0001). In contrast, the effective dose of sumatriptan (1.2 mg/kg, i.p.) and coadministration of subeffective doses of sumatriptan (0.3 mg/kg) + 7-NI (30 mg/kg) significantly reversed the PTZ-induced overexpression of nNOS (*P* ≤ 0.01, *P* ≤ 0.001, respectively) gene. 

**Figure 10:**
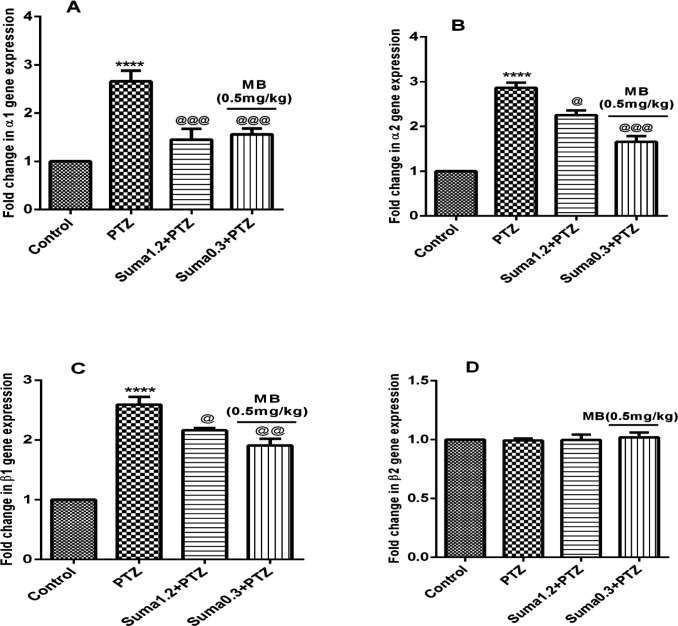
Effect of sumatriptan on relative mRNA expression of (A) α1*,* (B) α2*,* (C) β1*, *and (D) β2 subunits of soluble guanylyl cyclase genes in cerebral cortex of mices against PTZ-induced clonic seizure. Data are expressed as mean ± S.E.M. for 3 mices, analyzed by one-way ANOVA followed by Tukey's *post-hoc *test. ****P ≤ 0.0001 compared to vehicle control, ^@^*P* ≤ 0.05, ^@@^*P* ≤ 0.01, ^@@@^*P* ≤ 0.001 compared to PTZ group

Moreover, the effective dose of sumatriptan significantly downregulated the PTZ-induced overexpression of α1 (*P* ≤ 0.001, Figure 10A), α2 (*P* ≤ 0.05, Figure 10B), and β1 (*P* ≤ 0.05, Figure 10C) genes compared to the PTZ treated group. However, coadministration of subeffective doses of sumatriptan (0.3 mg/kg, i.p.) + MB (0.5 mg/kg, i.p.) significantly downregulated the PTZ-induced overexpression of α1 (*P* ≤ 0.001, Figure 10A), α2 (*P *≤ 0.001, Figure 10B), and β1 (*P* ≤ 0.01, Figure 10C) genes compared to PTZ-treated group. In contrast, the gene expression of β2 remained non-significant (*P* > 0.05) in all four groups.

## Discussion

In the present study, we examined the possible role of NO-cGMP signaling pathway in anticonvulsant effect of acute sumatriptan administration using specific and nonspecific inhibitors of NOS, sGC, and PDE5 in mices as involvement of NO-cGMP pathway in PTZ-induced seizure has been reported recently (27). In addition, we also evaluated the contribution of inducible NOS (iNOS) in this effect. Furthermore, all findings were confirmed by studying the mRNA expression of nNOS, α1, α2, β1, and β2 genes by qRT-PCR analysis of the mices cerebral cortex tissues. 

Epileptic seizure is phenomenon of excessive and hyper synchronous discharge of a set of neurons in brain as result of neuronal excitation due to Ca^2+^ influx (28). Regardless of the manufacture of countless anticonvuulsive therapeutic options, newer drugs with more potent antiepileptic activity and fewer side effects are needed to explore (29). Sumatriptan is a selective agonist of 5-HT1B/1D autoreceptors on serotonergic terminals. Despite of major clinical application of sumatriptan in migraine, neuroprotective effect of this drug in various studies including cerebral ischemia, tolerance and dependence, seizure, depression and obsessive-compulsive disorder (OCD), and anxiety has been reported recently (15, 21, 30-32).

Nitric oxide (NO) is an important intracellular signaling molecule, produced from three different isoforms of NOS through activation of L-arginine depending upon intracellular pathophysiological processes (33). Specifically in brain, the increased intracellular influx of Са^2+ ^initiates NOS stimulation specifically nNOS and subsequent increase in NO concentration (34). The contribution of nNOS in neuronal disorders especially in modulation of seizure susceptibility is well-established (35).

The involvement of nitrergic system in PTZ-induced seizure is evident from a number of previous studies (23, 36). Moreover, the role of NOS inhibitors as anticonvulsant agents through diminution of NO concentration against PTZ-induced seizure is well reported in literature (37-39). Furthermore, the anticonvulsant properties of sumatriptan against PTZ-induced seizure have been already studied (40). In addition, a number of studies reported the involvement of NO as a main signaling mechanism underlying the therapeutic and pharmacological effects of sumatriptan (17, 18, 41). As shown in Figure 3, the subeffective doses of L-NNA and 7-NI augmented the anticonvulsive effect of subeffective administration of sumatriptan. This data reveals the involvement of NO in anticonvulsant effect of sumatriptan against PTZ-induced seizure, which corroborates the previous experiments (21).

It has been demonstrated that enhanced excitatory neurotransmission by NO lead to sGC stimulation and subsequent activation of cGMP in post synaptic membranes (42). Furthermore, the functional stimulation of cGMP leads to neurodegenerative disorders including epilepsy (43). Evidences have been shown the involvement of NO and cGMP pathways in therapeutic effects of sumatriptan. It has been reported that during migraine episode, the NO-mediated increased levels of cGMP were significantly reversed by sumatriptan in L-arginine-NO-cGMP dependent manner (16). As shows in Figure 5, in consistence with previous studies, the subeffective dose of sumatriptan when coadministered with cGMP inhibitors significantly attenuated the PTZ-induced seizure in mice (13). In addition, as shown in Figure 7, sumatriptan significantly reduced the nitrite concentration in PFC of mice, which strengthens the involvement of NO-cGMP pathway in anticonvulsant activity of sumatriptan as reported previously (44).

The sGC comprises of two subunits of α and β represents in four isoform namely α1, α2, β1, and β2, and exists in two heterodimers of α1/β1 and α2/β1, whereas, the homodimers (β2) are enzymatically inactive (45). The activation of sGC subunits leads to cGMP simulation (10, 11). The NO-mediated neuronal excitability in cerebral cortex is well-known (46). Moreover, in cerebral cortex, the sGC subunits present in form of two functional heterodimers of α1/β1 and α2/β1, which coexist with nNOS (9, 47). Based on these evidences, as shown in Figures 9 and 10, the results of the present study showed that sumatriptan downregulated the PTZ-induced mRNA expression of nNOS, and α1, α2 and β1 subunits of soluble guanylyl cyclase as reported previously (48, 49).

**Table 1:** Nucleotide sequences of the primers used in study

**Gene name**	**Forward primer**	**Reverse primer**	**Amplicon length**
nNOS	AATGGGTCTTGTGTATGCTAGG	ATGAAGATGGGAAGGAGTTGG	186
α1	GTAAGTGATAGCGGTGCCC	ACAGTGATCTTGCTTCCCAG	130
α2	TGCACAGACACTTAAGGAGAAG	CTAGCATCCTGGTCTTGTGATC	158
β1	TGAGATGCAGAAACAAGCCC	GAACCCAGAATCCCCAGAAG	167
β2	CACTGTATCCTCTGATCTCTGC	AAATCTCACCATCGTACCTGC	157
GAPDH	AATACGGCTACAGCAACAGG	TGGGATGGAAATTGTGAGGG	160

## Conclusion

The findings of the present study demonstrated that acute administration of sumatriptan reversed the PTZ-induced seizure at least, in part, through inhibition of NO-cGMP signaling pathway. 
